# Ultrasound Radiomics in Pediatric Imaging: Current Applications, Challenges, and Future Directions Toward Clinical Implementation

**DOI:** 10.3390/diagnostics16111669

**Published:** 2026-05-28

**Authors:** Maria Mezher, Mohannad Elgamal, Sean Schoeman, Maryam Al-Hasani, Hansel J. Otero, Laith R. Sultan

**Affiliations:** 1 Radiology Department, Children’s Hospital of Philadelphia, 3401 Civic Center Blvd., Philadelphia, PA 19146, USA; mezherm@chop.edu (M.M.); elgamalm@chop.edu (M.E.); schoemans@chop.edu (S.S.); oteroh@chop.edu (H.J.O.); 2 Perelman School of Medicine, University of Pennsylvania, Philadelphia, PA 19104, USA

**Keywords:** pediatric imaging, ultrasound, radiomics, quantitative analysis, image segmentation, tissue characterization, imaging workflow, artificial intelligence, machine learning

## Abstract

Ultrasound is widely used in pediatric imaging because it is safe, portable, real-time, and free of ionizing radiation, but interpretation remains qualitative and operator-dependent. Ultrasound radiomics can extract quantitative features from standard grayscale images, providing potential biomarkers of tissue patterns not readily apparent on visual assessment. This structured narrative review summarizes pediatric ultrasound radiomics applications across organ systems and key barriers to clinical translation. Current evidence suggests promise in neonatal brain injury, neurodevelopmental assessment, liver disease staging, renal characterization, oncologic risk stratification, and emerging lung and muscle applications. However, most studies remain limited by retrospective single-center designs, small cohorts, acquisition variability, segmentation inconsistency, and limited external validation. Future progress will require standardized workflows, prospective multicenter validation, and clinically interpretable integration into decision-support systems.

## 1. Introduction

Over recent years, ultrasound has become a primary imaging modality in pediatrics because of its safety, accessibility, portability, low cost, real-time capability, and lack of ionizing radiation [1,2,3,4]. These advantages are particularly important in children, who may require repeated imaging and who face unique challenges including heightened radiation sensitivity, the potential need for sedation during longer examinations such as magnetic resonance imaging, small body size, and age-dependent anatomy [2]. These factors have reinforced ultrasound’s central role in pediatric diagnostic workflows, particularly for bedside imaging, longitudinal assessment, and evaluation of medically vulnerable patients [5,6].

Despite this versatility, conventional ultrasound interpretation remains largely qualitative and operator-dependent, with image acquisition and reporting influenced by sonographer technique, scanner settings, acoustic windows, and radiologist experience [7,8]. This subjectivity limits reproducibility across institutions and over time, particularly when subtle parenchymal or treatment-related tissue changes fall below the threshold of visual detectability on routine grayscale imaging [9,10]. These limitations have driven growing interest in radiomics—the extraction of quantitative imaging features from medical images to investigate associations with clinical, biological, or outcome-based information [11,12,13,14,15,16,17,18]. Applied to ultrasound, radiomics converts grayscale pixel data into numerical descriptors of signal intensity, spatial texture, morphology, and heterogeneity, capturing tissue patterns not readily appreciable by visual assessment alone and potentially supporting disease characterization, risk stratification, treatment monitoring, and outcome prediction [19,20,21].

The application of radiomics to pediatric ultrasound is a natural extension of this framework, given that ultrasound already underpins bedside evaluation, longitudinal follow-up, and disease surveillance in children. Current studies suggest potential applications in neonatal brain imaging, liver disease, renal characterization, oncologic risk stratification, lung disease, and muscle assessment. However, pediatric ultrasound radiomics remains an emerging research area rather than a routine clinical tool. Evidence is limited by small cohort sizes, single-center study designs, heterogeneous acquisition protocols, variable segmentation methods, limited reproducibility assessment, and insufficient external validation. Ultrasound radiomics also require particular methodological caution because image appearance is highly sensitive to acquisition parameters, scanner vendor, operator technique, speckle, artifacts, patient motion, and segmentation variability [22,23,24,25,26,27,28,29].

Unlike prior reviews that have largely addressed radiomics broadly or adult-dominant ultrasound applications, this structured narrative review aims to evaluate the current applications of radiomics in pediatric ultrasound imaging across organ systems and disease categories; identify methodological limitations and translational challenges that constrain clinical adoption; and outline a practical path toward reproducible, validated, and clinically meaningful ultrasound radiomic biomarkers in pediatric radiology [22,30].

## 2. Overview of Ultrasound Radiomics

This section provides a brief overview of the ultrasound radiomics workflow and major feature categories needed to interpret the current literature.

### 2.1. Radiomics Workflow in Ultrasound

**Figure 1:**
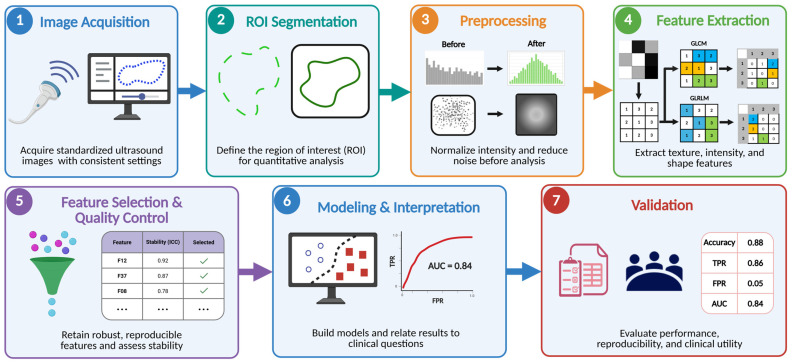
Created in BioRender. Schoeman, S. (2026) https://BioRender.com/suzbpzt, accessed on 24 May 2026. Schematic showing the steps of pediatric ultrasound radiomics workflow. Standardized US images are first acquired using consistent imaging parameters (1), followed by manual or semi-automated segmentation of the region of interest (ROI) corresponding to the target lesion or tissue (2). Preprocessing techniques including intensity normalization and denoising are then applied to enhance feature reproducibility prior to analysis (3). Radiomic features are then extracted from the ROI (4), these include higher-order texture features derived from matrices such as the gray-level co-occurrence matrix (GLCM) and gray-level run-length matrix (GLRM). In panel 4, the grayscale squares represent discretized pixel intensity values within the ROI, while the colored squares illustrate quantified spatial relationships between neighboring gray levels. These values correspond to frequency or probability of pixel intensity occurrences and are subsequently used to calculate texture metrics. Following extraction, redundant or unstable features are removed through quality control processes (5). Finally, after modeling is applied to associate features to relevant outcomes (6), final model performance is evaluated using metrics such as true or false positive rate and receiver operating characteristic curve (AUC) (7).

The ultrasound radiomics workflow generally includes image acquisition, region-of-interest segmentation, preprocessing, feature extraction, feature selection, model development, and validation [Figure 1]. Each step can influence the final radiomic output and should be carefully considered in pediatric ultrasound studies.

The first step is image acquisition. Image quality and feature values may be affected by scanner type, transducer frequency, gain, depth, focal zone placement, dynamic range, reconstruction parameters, and operator technique [22]. For ultrasound radiomics, standardization of acquisition protocols is particularly important because radiomic features are highly sensitive to technical variability.

The second step is region-of-interest segmentation, in which the target tissue or lesion is delineated manually, semiautomatically, or automatically [23]. Accurate segmentation is critical because extracted radiomic features are directly dependent on the selected region of interest. Inconsistent boundary definition may substantially affect feature reproducibility and model performance, particularly in pediatric imaging, where small anatomy, motion, variable acoustic windows, and subtle tissue boundaries can make segmentation challenging.

Preprocessing is then performed to improve image consistency before feature extraction. Common preprocessing steps include resampling, intensity normalization, gray-level discretization, denoising, and image filtering [24]. These steps are intended to reduce nonbiological variation and improve feature robustness, although excessive preprocessing may alter biologically meaningful image information. Standardization efforts such as the Image Biomarker Standardization Initiative have emphasized the importance of harmonized preprocessing and feature definitions to improve reproducibility across studies and software platforms.

After preprocessing, feature extraction is performed using dedicated software platforms such as PyRadiomics, MaZda, or other validated feature-extraction tools [25,26,31]. These tools can compute a broad range of engineered features from the segmented region of interest, including first-order, texture, shape, and filtered features. Because radiomics datasets often contain a large number of potentially redundant variables, feature selection is usually required to reduce dimensionality and retain the most informative and stable features. Common approaches include statistical filtering, reproducibility testing, correlation analysis, and regularization methods such as least absolute shrinkage and selection operator [16,27,28,29]. Selected features may then be incorporated into statistical or machine learning models for classification, prediction, or outcome assessment. Proper model development requires internal validation and, ideally, external testing to assess generalizability.

### 2.2. Radiomic Feature Categories

**Figure 2:**
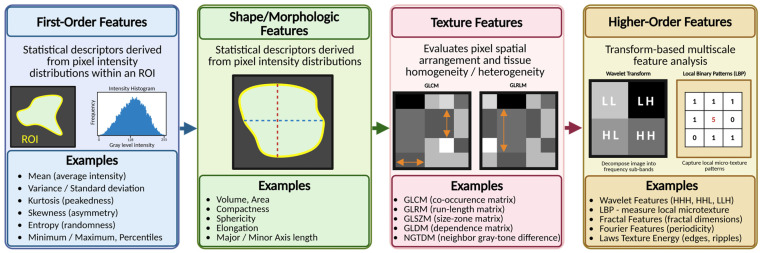
Created in BioRender. Schoeman, S. (2026) https://BioRender.com/n2plbd6, accessed on 24 May 2026. Figure showing the categories of radiomics features studied in pediatric ultrasound imaging. First-order features quantify the distribution of pixel intensities within the region of interest (ROI) without considering spatial relationships. Shape and morphologic features describe ROI geometry and size characteristics. Texture features evaluate spatial relationships between neighboring pixel intensities using various matrices. The arrows indicate spatial relationships and directional comparisons used to construct texture matrices such as GLCM and GLRM. Higher-order features apply mathematical transformations: the gray scale box represents wavelet-decomposed image sub-bands capturing image information at different spatial frequencies and orientations (e.g., low-low [LL], low-high [LH] frequency components). The numerical grid illustrates transformed pixel pattern (such as LBP) encoding used to quantify fine-scale texture characteristics.

Radiomic features are commonly grouped into first-order, texture, shape, and higher-order features according to the type of image information they describe [Figure 2] [32].

First-order features are statistical descriptors derived from the distribution of pixel intensities within the region of interest, without accounting for spatial relationships. Common examples include mean, variance, skewness, kurtosis, and entropy. These features characterize the overall grayscale intensity profile of the tissue and can reflect signal magnitude, dispersion, asymmetry, peakedness, and randomness.

Texture features quantify spatial variation and gray-level arrangement within the image and are often useful when tissue heterogeneity is of interest. One of the most widely used texture approaches is the gray-level co-occurrence matrix, which evaluates how often pairs of gray levels occur at a defined spatial relationship and captures local spatial organization [32,33]. Common gray-level co-occurrence matrix features include contrast, correlation, homogeneity, and entropy. Another important texture family is the gray-level run-length matrix, which captures the length of consecutive runs of pixels with the same gray level in a specified direction. Common gray-level run-length matrix features include short-run emphasis, long-run emphasis, and gray-level nonuniformity.

Shape features describe the geometry of a segmented lesion or structure, including size, contour, compactness, and irregularity. Although shape features are commonly used in CT and MRI radiomics, their use in ultrasound may be more limited because lesion and tissue boundaries are often affected by acoustic window, speckle, shadowing, and segmentation variability.

Higher-order features are generated after applying mathematical filters or transforms to the original image to emphasize patterns at different scales or frequencies [34]. Common examples include wavelet features, which decompose the image into multiscale frequency sub-bands, as well as fractal dimension and local binary pattern descriptors, which may capture structural complexity and local microtexture. These approaches may enhance sensitivity to fine-scale heterogeneity but can also be sensitive to preprocessing choices and image quality.

Figure 2 shows the major categories of radiomic features in pediatric ultrasound imaging. Radiomic features include first-order intensity features, texture features, shape features, and higher-order filtered features. These categories provide complementary information about tissue intensity, spatial organization, morphology, and multiscale heterogeneity.

### 2.3. Ultrasound-Specific Considerations

Although the general radiomics workflow is similar across imaging modalities, ultrasound radiomics requires special consideration. Unlike CT and MRI, ultrasound does not have a standardized grayscale intensity scale, and image appearance is strongly influenced by acquisition settings, scanner vendor, probe selection, transducer pressure, patient motion, speckle, attenuation, and artifacts. These factors may affect radiomic feature stability and reduce reproducibility across scanners, operators, institutions, and patient populations.

These challenges are especially important in pediatric imaging, where small anatomy, developmental variation, limited patient cooperation, and disease rarity may further complicate image acquisition and model validation. Therefore, pediatric ultrasound radiomics should be interpreted as an emerging quantitative research approach that requires careful attention to acquisition standardization, segmentation reliability, feature reproducibility, and external validation before routine clinical implementation.

## 3. Technical Challenges Unique to Ultrasound Radiomics

Despite its promise as a noninvasive biomarker platform, ultrasound radiomics remain particularly vulnerable to technical variability across the imaging and analytic pipeline. In contrast to CT and MRI, ultrasound is intrinsically operator-dependent, and image appearance is strongly influenced by acquisition settings and system-specific factors. As a result, the reproducibility of radiomic features can be more difficult to ensure, which remains a central barrier to robust model development and clinical translation [22,35,36,37].

### 3.1. Acquisition-Related Variability

A major limitation of ultrasound radiomics is the variability introduced during image acquisition. Probe frequency, focal depth, gain, dynamic range, transducer pressure, scanner manufacturer, and operator technique can all alter image appearance, even when imaging the same tissue target [35,37,38]. This dependence on acquisition conditions is especially problematic for radiomics because quantitative features may capture not only underlying tissue characteristics but also technical differences in image formation.

In a reproducibility study using phantom and clinical ultrasound data, only a minority of extracted ultrasound radiomic features remained stable across changes in machine, segmentation location, and feature-extraction platform, underscoring the sensitivity of many features to acquisition-related variation [37]. These findings highlight the need for standardized acquisition protocols and careful documentation of scanner settings in ultrasound radiomics studies.

### 3.2. Preprocessing and Normalization

Because ultrasound grayscale values are highly system-dependent, preprocessing and normalization are often required to improve comparability across images, scanners, and institutions [22,35,39]. However, unlike CT, where intensity values have a more standardized physical basis, ultrasound lacks a universal grayscale intensity scale. Therefore, preprocessing choices such as resampling, discretization, filtering, contrast enhancement, speckle reduction, and intensity normalization may substantially influence downstream radiomic features [22,39,40].

Accordingly, normalization should not be considered a simple technical adjustment. Instead, it represents a critical methodological step that must be explicitly defined, justified, and validated within each ultrasound radiomics workflow. Without transparent preprocessing strategies, radiomic models may inadvertently learn scanner- or protocol-specific differences rather than biologically meaningful imaging phenotypes [22,39,40].

### 3.3. Radiomic Feature Reproducibility and Robustness

Reproducibility is a central requirement for any radiomic biomarker intended for clinical translation. In ultrasound radiomics, feature reproducibility can be affected by multiple interacting factors, including scanner vendor, acquisition settings, operator technique, image quality, preprocessing strategy, segmentation approach, and feature-extraction software. In a dedicated ultrasound radiomics reproducibility study, only a minority of extracted features remained reproducible when ultrasound machine, segmentation location, or feature-extraction platform changed, underscoring the sensitivity of ultrasound-derived features to technical variation [37].

For this reason, reproducibility assessment should be incorporated early in ultrasound radiomics studies rather than treated as a secondary validation step. Features should be evaluated for stability across repeated acquisitions, different observers, alternative segmentations, preprocessing methods, and, when possible, different ultrasound platforms. Common approaches include intraclass correlation coefficients, test–retest analysis, interobserver and intraobserver agreement, phantom-based reproducibility testing, and image perturbation analysis [41,42,43]. These methods can help identify robust features that are less sensitive to technical variation and more likely to generalize across clinical settings. This is particularly important in pediatric ultrasound, where small anatomy, motion, variable acoustic windows, and age-related developmental changes may further affect image appearance. Prioritizing reproducible features may reduce overfitting, improve model generalizability, and increase the likelihood that ultrasound radiomics can evolve from exploratory research toward clinically reliable imaging biomarkers.

### 3.4. Speckle Noise and Image Artifacts

**Figure 3:**
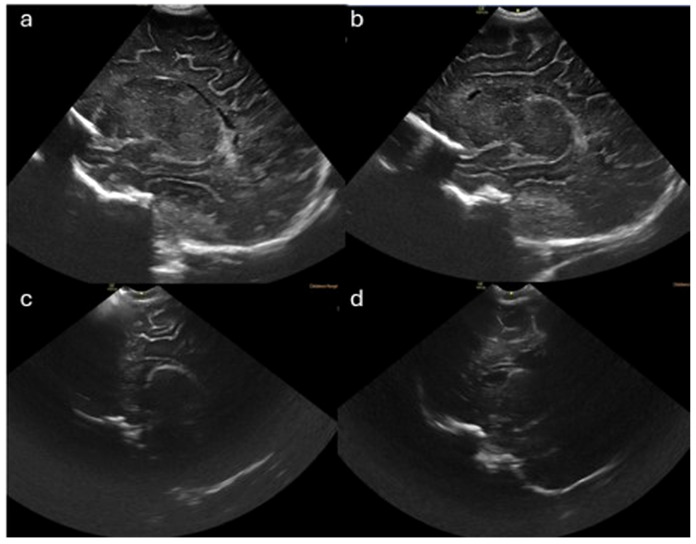
Influence of image quality on radiomics analysis. Representative cranial ultrasound images are shown to illustrate the effect of image quality on radiomic assessment. The upper panel (**a**,**b**) includes good quality images with adequate visualization of cerebral parenchyma, enabling reliable segmentation and feature extraction. The lower panel (**c**,**d**) demonstrates images degraded by artifacts and noise, which obscure parenchymal texture and may reduce the robustness and reproducibility of radiomic measurements. This figure underscores the importance of rigorous image quality assurance in brain ultrasound radiomics workflows.

Speckle is an inherent property of ultrasound imaging that arises from interference among subresolution scatterers and substantially influences image texture [44,45]. Although speckle may contain biologically relevant information in some quantitative ultrasound applications, it can also reduce contrast resolution, obscure lesion boundaries, and introduce stochastic texture patterns that confound feature stability [44,45,46]. Additional artifacts, including acoustic shadowing, posterior enhancement, reverberation, attenuation-related distortion, and motion, may further introduce nonbiologic heterogeneity into the image [45,46] [Figure 3]. Because radiomics is fundamentally designed to quantify image heterogeneity, these artifact-related patterns can be inadvertently encoded as discriminatory features unless careful quality control and preprocessing are applied [35,44,46].

### 3.5. Segmentation Variability and Automated Segmentation

Segmentation represents another major source of error in ultrasound radiomics. Manual delineation is still widely used, particularly in pediatric and research settings, yet ultrasound lesion margins are often indistinct because of low contrast, speckle, and artifact contamination [35,41,47]. Consequently, interobserver and intraobserver variability in region-of-interest definition can have a substantial effect on extracted features and on subsequent model performance [41,47,48]. Prior radiomics work has shown that feature robustness may decline meaningfully when segmentations vary across readers, and ultrasound-specific studies similarly suggest that automated or semiautomated approaches may improve reproducibility compared with fully manual workflows [37,41,47,48]. Even so, segmentation automation in ultrasound remains an evolving area and still requires rigorous validation before widespread adoption [37,41,42,43,47,48].

Deep learning-based segmentation methods provide a promising strategy to address this bottleneck. Convolutional neural network architectures, particularly U-Net and its variants, have become widely used for biomedical image segmentation and are increasingly being developed for automated and semiautomated ultrasound segmentation [49,50]. These models can learn spatial and contextual features from annotated imaging datasets and may help improve boundary consistency, reduce operator-dependent variability, and standardize region-of-interest placement across readers and institutions. This is particularly relevant in pediatric ultrasound, where small anatomy, motion, variable acoustic windows, and heterogeneous image quality can further complicate manual segmentation.

Despite this promise, automated ultrasound segmentation remains an evolving area. Deep learning models are sensitive to training data quality, annotation standards, scanner type, acquisition parameters, and patient population. Therefore, rigorous internal and external validation, quality-control procedures, and human oversight remain necessary before large scale clinical incorporation.

### 3.6. Model Calibration and Clinical Utility

Most pediatric ultrasound radiomics studies report discrimination metrics such as AUC, sensitivity, specificity, and accuracy. While these measures indicate how well a model separates disease categories or severity groups, they do not determine whether predicted probabilities are clinically reliable. A model may achieve a high AUC yet still overestimate or underestimate absolute disease risk, limiting its usefulness for individualized decision-making and clinical implementation [51,52].

Calibration, which reflects the agreement between predicted probabilities and observed outcomes, is therefore an important but underreported component of model evaluation. It can be assessed using calibration plots, calibration intercept and slope, and the Brier score [53,54]. In pediatric ultrasound radiomics, this is particularly relevant because proposed applications often involve risk stratification, biopsy selection, follow-up planning, treatment guidance, or prognostic counseling. Poorly calibrated models may appear statistically accurate while providing misleading risk estimates that could affect patient management.

Clinical utility should also be evaluated before radiomics models are considered for practice. Approaches such as decision curve analysis and net benefit assessment can determine whether a model improves decision-making compared with existing clinical pathways, expert interpretation, or default strategies [53]. Given the small datasets, low disease prevalence in some applications, and potential consequences of unnecessary imaging, biopsy, or treatment escalation in children, future pediatric ultrasound radiomics studies should move beyond AUC alone and report discrimination, calibration, and clinical utility to support clinically interpretable and actionable prediction models.

### 3.7. Small Pediatric Cohorts and Limited Generalizability

These technical challenges are amplified in pediatric imaging, where ultrasound radiomics studies are frequently constrained by small cohorts, single-center design, and limited external validation. Small datasets reduce statistical power, increase the risk of overfitting, and are particularly problematic in radiomics because the number of extracted features can vastly exceed the number of available subjects [36,55,56]. More broadly, recent evaluations of the ultrasound radiomics literature have repeatedly identified high risk of bias related to small sample size, restricted case diversity, and lack of multicenter validation [55,56]. In pediatric applications, these issues are even more pronounced because of disease rarity, heterogeneous age- and development-related anatomy, and practical barriers to assembling large, harmonized datasets [22,35,36,39,57].

### 3.8. Need for End-to-End Standardization

Ultimately, the most important barrier to clinical translation is the lack of end-to-end standardization. Reliable ultrasound radiomics requires standardized acquisition protocols, transparent preprocessing workflows, reproducible feature definitions, robust segmentation methods, quality-control procedures, and external multicenter validation [23,35,36,39,49].

The Image Biomarker Standardization Initiative has substantially improved harmonization of radiomic feature definitions and benchmarking across software platforms, but standardization of the broader ultrasound workflow remains incomplete, particularly at the levels of acquisition, preprocessing, and image quality control [23,39]. Reporting frameworks such as CLAIM and CLEAR further emphasize that methodological transparency, reproducibility assessment, and validation are essential for trustworthy imaging AI and radiomics research [57,58]. Without these measures, the apparent diagnostic performance of ultrasound radiomics models may not translate into reliable clinical performance across scanners, operators, institutions, or pediatric patient populations.

## 4. Review Design

This review was conducted as a structured narrative review of the pediatric ultrasound (US) radiomics literature. Given the heterogeneity in the existing literature, regarding study design, organ/tissue systems, image acquisition technique, segmentation methodology, feature extraction pipelines, and outcome measures, a formal meta-analysis was not feasible. The review aimed to synthesize current applications of US radiomics within the sphere of pediatric imaging while critically evaluating each study’s methodologic quality, reproducibility, and translational readiness.

### 4.1. Literature Search Strategy

A literature search was performed using PubMed, Scopus, and Google Scholar databases for studies published through March 2026. Search terms included combinations of: “ultrasound radiomics”, “quantitative ultrasound”, “ultrasound texture analysis”, “pediatric”, “children”, and organ-specific/pathology terms (i.e., brain, renal, tumor, musculoskeletal).

### 4.2. Eligibility Criteria

Studies were included if:

- The cohort included pediatric patients (<18 years);
- They applied grayscale US-based radiomic, texture, or quantitative feature extraction methodologies;
- They evaluated diagnostic, prognostic, predictive, or classification performance;
- They included original research data;
- They were published in English.

Studies were excluded that:

- Exclusively included adult (>18 years) subjects;
- Did not apply quantitative feature extraction based on grayscale US image information including other US modalities, e.g., Doppler parameters and advanced ultrasound techniques (e.g., elastography, CEUS) were not included or critically assessed.
- Were not clinical or translational research (review articles, editorials, case series or reports);
- Analyzed non-US imaging modalities (CT or MRI);
- Did not clearly define methodology employed for radiomic analysis (feature extraction or model development);

Study Selection and Data Extraction
Eligible studies were reviewed and categorized by primary clinical applications. This included:

- Brain imaging;
- Liver imaging;
- Renal Imaging;
- Oncologic Imaging;
- Emerging areas—including lung and musculoskeletal.

The following variables were analyzed, when available, to enhance review uniformity:

- Study design, sample size, clinical application, and patient population;
- Ultrasound modality and acquisition parameters;
- Segmentation methodology and radiomic pipeline;
- Radiomic features extracted and models used;
- Study limitations including validation technique or image acquisition methods;
- Level of evidence (per Oxford Center for Evidence-Based Medicine guidelines) [59].

### 4.3. Evidence Synthesis

Given the substantial heterogeneity within the growing body of research on pediatric-specific US-radiomics application, findings were synthesized qualitatively, rather than quantitatively pooled. The literature was interpreted with primary emphasis on recurring methodological limitations and emerging/potential areas of promise relevant to the scope of pediatric imaging applications.

## 5. Current Clinical Applications of Ultrasound Radiomics in Pediatrics

Because detailed study-level methodology, including segmentation approach, feature classes, software, model type, validation strategy, performance metrics, limitations, and evidence level, are summarized in Table 1, this section focuses on the clinical task, principal findings, critical interpretation, and translational readiness of pediatric ultrasound radiomics applications.

The volume and maturity of available evidence differ substantially across organ systems; therefore, the depth of discussion varies accordingly. Neonatal brain imaging is discussed in greater detail because it currently represents the largest and most developed pediatric ultrasound radiomics literature, whereas liver, renal, oncologic, lung, and musculoskeletal applications remain more limited or emerging. To improve readability, common limitations, including small sample size, single-center design, heterogeneous acquisition, variable segmentation, and limited external validation, are considered collectively in this section and revisited in the future directions rather than repeated in detail for each application.

### 5.1. Brain Imaging

Neonatal brain imaging currently represents the most developed pediatric application of ultrasound radiomics, but the evidence remains stronger as proof-of-concept than as a clinically deployable tool. Across studies, radiomics has shown that visually subtle or diffuse white matter abnormalities can be quantified from routine cranial ultrasound images. This is clinically meaningful because conventional neurosonography is limited in detecting non-cystic or evolving injury patterns.

#### 5.1.1. Preterm White Matter Injury and Early Detection

Narchi et al. evaluated 20 very-low-birth-weight neonates and showed that texture analysis of initial cranial ultrasound could distinguish transient periventricular echogenicity from echogenicity that later progressed to cystic periventricular leukomalacia (PVL), with AUC values of 0.98 and 0.92 in sagittal and coronal planes, respectively [60]. Although promising, this study is best interpreted as an early proof-of-concept because of its small retrospective cohort, limited follow-up, and lack of external validation.

You et al. evaluated 33 preterm neonates and linked increased textural heterogeneity with MRI-graded severe white matter injury (WMI), with entropy achieving an AUC of 0.865 [61]. This strengthened the biologic plausibility that radiomic features may reflect structural disorganization in injured white matter. However, the method did not reliably distinguish normal from mild WMI, which is a critical limitation because subtle non-cystic injury is often the most difficult and clinically relevant target.

Zhu et al. advanced the field by testing higher-dimensional and automated radiomics approaches for WMI detection in 158 preterm neonates, with reported AUC values of around 0.85 [62]. A later multiplanar study in 267 premature infants achieved a validation AUC of 0.91, suggesting that multi-plane radiomic assessment may better capture the spatial heterogeneity of WMI than single-plane analysis [62,63]. These studies are more methodologically mature than earlier texture-analysis work, but their clinical readiness remains limited by single-center development and incomplete external validation.

#### 5.1.2. Longitudinal Monitoring and Neurodevelopmental Risk

Longitudinal monitoring may be one of the most clinically compelling directions for neonatal brain radiomics. Jung et al. showed that serial texture change helped distinguish evolving periventricular leukomalacia from resolving periventricular echogenicity, with the strongest longitudinal measure achieving an AUC of 0.82 [64]. Laccetta et al. also found that quantitative grayscale measures of periventricular white matter were associated with later neurodevelopmental outcomes [65]. These findings suggest that texture trajectories may provide more clinically meaningful information than single-time-point assessment, although longer-term outcome validation remains needed.

#### 5.1.3. Expanding Applications Beyond Preterm Injury

The application of brain ultrasound radiomics to full-term exposure-related conditions is promising but remains exploratory. Zimina et al. studied 89 full-term newborns with diabetic fetopathy and reported that radiomic models could detect brain differences not apparent on conventional neurosonography, with the best regional model achieving an AUC of 0.85 [66]. Sultan et al. applied neonatal brain ultrasound radiomics to HIV-exposed uninfected and HIV-unexposed newborns, reporting AUC values of 0.72 in the basal ganglia and 0.76 in the periventricular white matter [67]. These studies broaden the potential role of ultrasound radiomics beyond preterm injury, particularly in settings where MRI may be unavailable.

Overall, neonatal brain ultrasound radiomics has progressed from early texture analysis to higher-dimensional modeling, multiplanar assessment, longitudinal monitoring, and automated segmentation. Its main promise lies in detecting subtle or diffuse tissue abnormalities that are visually inapparent or difficult to grade reproducibly. Future studies should determine whether radiomics improves risk stratification, monitoring, or decision-making beyond expert cranial ultrasound and whether radiomic markers correlate with longitudinal neurodevelopmental outcomes.

### 5.2. Liver Imaging

Compared with neonatal brain imaging, pediatric liver ultrasound radiomics is less developed but addresses an important clinical gap. Conventional ultrasound is widely used for screening and follow-up of diffuse liver disease, yet visual assessment of echogenicity and attenuation remains subjective and insensitive to early or mild disease [68,69,70]. Radiomics may improve objectivity, but current pediatric evidence is narrow and largely focused on steatosis.

#### 5.2.1. Steatosis and Diffuse Liver Disease

The strongest pediatric evidence comes from Das et al., who evaluated 93 healthy controls and 39 children with NAFLD and showed that ultrasound texture analysis differentiated fatty liver disease from healthy liver tissue with an external validation AUC of 0.92 [71]. The model also outperformed simpler echogenicity-based measures such as the hepatorenal index, suggesting that radiomics may capture parenchymal fat-related texture changes beyond subjective grayscale interpretation.

However, the clinical role of this approach remains uncertain. Pediatric NAFLD/MASLD exists across a spectrum of fat burden, inflammation, fibrosis, and metabolic risk. Therefore, binary classification against healthy controls may not reflect the more difficult clinical tasks of grading disease severity, detecting early disease, or monitoring treatment response. Future studies should test whether radiomics adds value over MRI-PDFF, elastography, laboratory markers, and clinical risk scores.

#### 5.2.2. Fibrosis and Pediatric-Specific Barriers

Pediatric fibrosis is an important potential application but remains under-validated. Adult studies suggest that ultrasound radiomics may capture fibrotic architectural distortion, but extrapolation to children is problematic [72,73]. Pediatric liver texture is influenced by growth, body habitus, inflammation, congestion, underlying diagnosis, and treatment status. Without age-specific reference ranges and disease-specific validation, radiomic features may reflect developmental or technical variation rather than fibrosis.

#### 5.2.3. Critical Summary

The main promise of pediatric liver radiomics is improved objectivity in a modality already used widely for screening and follow-up. The next stage should focus on multicenter pediatric cohorts, standardized acquisition, age-adjusted normative data, and clinically relevant endpoints such as fibrosis stage, steatosis severity, progression, and treatment response.

### 5.3. Renal Imaging

Renal ultrasound radiomics is one of the more clinically plausible pediatric applications because ultrasound is already central to renal evaluation, and several renal diseases have functional, laboratory, or histologic reference standards. The key question is whether radiomics can improve upon subjective echogenicity, which is commonly used but poorly specific.

#### 5.3.1. Parenchymal Disease Characterization

**Figure 4:**
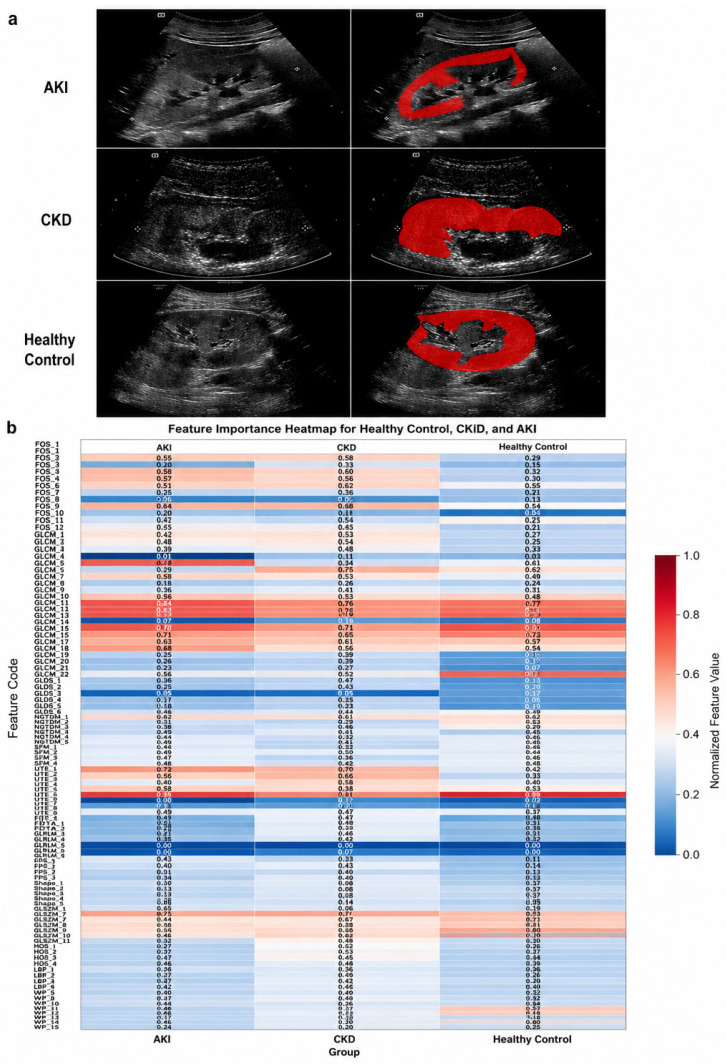
Radiomics reveal quantitative renal texture differences beyond visual grayscale assessment. (**a**) Representative grayscale renal ultrasound images from CKD, AKI, and healthy control cases show broadly overlapping visual appearances, underscoring the difficulty of distinguishing these groups by routine inspection alone. In contrast, normalized radiomic feature profiling (**b**) demonstrates distinct texture signatures across disease states. This example illustrates the potential of ultrasound radiomics to uncover subtle parenchymal differences not readily visible on conventional imaging. This figure was adapted from De Leon-Benedetti et al. [26].

De Leon-Benedetti et al. evaluated 31 pediatric subjects with AKI, CKD, or healthy kidneys and reported that radiomics models achieved an accuracy of around 0.90 for group differentiation [Figure 4] [25]. The observed pattern of greater homogeneity in AKI and greater heterogeneity in CKD is biologically plausible and clinically relevant. This study supports the potential of radiomics for quantitative renal parenchymal characterization.

#### 5.3.2. Glomerulonephropathy and Histologic Risk Stratification

Radiomics studies in biopsy-confirmed glomerulonephropathy are particularly important because pathology provides a strong reference standard. Kou et al. studied 313 biopsy-confirmed pediatric glomerulonephropathy cases and reported high validation AUCs for differentiating IgA nephropathy, minimal change disease, and Henoch–Schonlein purpura nephritis, ranging from 0.91 to 0.98 [74]. Chen et al. evaluated 440 biopsy-confirmed cases of Henoch–Schonlein purpura nephritis and reported a validation AUC of 0.87 for predicting histologic severity, particularly crescentic disease [75].

These results are promising, but they must be interpreted cautiously. High performance in single-center retrospective datasets may reflect disease selection, acquisition consistency, or overfitting rather than generalizable disease biology. The clinical value of renal radiomics will depend on whether it can predict histologic severity or disease progression in external cohorts and whether it changes decisions about biopsy, treatment intensity, or monitoring.

#### 5.3.3. Hydronephrosis and Longitudinal Monitoring

Sloan et al. studied 90 pediatric patients with hydronephrosis and achieved an AUC of 0.86 for differentiating low- from high-grade disease [76]. This is a practical application because hydronephrosis grading is routine but can be subjective. However, the incremental value over expert interpretation and standardized grading systems still needs clarification.

**Figure 5:**
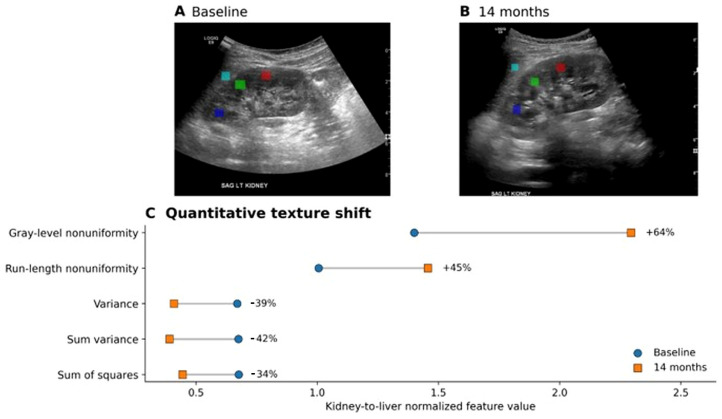
Example of radiomics-derived detection of subtle interval renal change beyond visual grayscale assessment. Baseline pre-angioplasty (**A**) and 14-month follow-up (**B**) grayscale renal ultrasound images appear broadly similar on conventional visual inspection. In contrast, quantitative texture analysis (**C**), based on renal cortical radiomic features normalized to a splenic reference region, demonstrates measurable longitudinal shifts in multiple texture parameters. This representative case illustrates the potential of ultrasound radiomics to identify subtle parenchymal changes not readily visible on standard grayscale imaging.

Early post-angioplasty radiomics work by Schoeman et al. suggests that texture analysis may detect longitudinal renal parenchymal changes not evident visually [Figure 5] [77]. This direction is attractive because radiomics may be most useful as a monitoring biomarker. However, such changes must be correlated with perfusion, renal function, blood pressure response, and clinical outcomes before they can be interpreted as meaningful recovery or remodeling.

#### 5.3.4. Critical Summary

Renal ultrasound radiomics has strong translational rationale, but the field remains immature. The most important future step is to demonstrate incremental value beyond conventional ultrasound, laboratory tests, pathology, and clinical variables. Multicenter validation, patient-level splitting, standardized cortical and medullary ROI definitions, and outcome-based endpoints will be essential.

A review of the application of US radiomics in pediatric renal imaging has demonstrated promising results in the relatively small studies in which it has been explored. Future research is needed to validate these initial findings in order to transition this research from ‘pilot studies’ in limited samples to validated results with relevant clinical use cases.

### 5.4. Oncologic Imaging

Pediatric oncologic ultrasound radiomics is promising because many tumors have pathologic or molecular reference standards, but current evidence remains in its early stages. The potential value of radiomics is not in replacing biopsy or pathology, but in improving noninvasive risk stratification, preoperative planning, and lesion characterization.

#### 5.4.1. Tumor Characterization and Prognostic Stratification

Zhu et al. evaluated 73 pediatric patients with peripheral neuroblastic tumors and reported strong differentiation of prognostic subsets, with a radiomics-only validation AUC of 0.918 [78]. This suggests that grayscale tumor texture may reflect biologically meaningful heterogeneity. These findings suggest that grayscale tumor texture may reflect biologically meaningful heterogeneity and could complement established clinical, histologic, and molecular risk factors.

#### 5.4.2. Thyroid and Thoracic Applications

Li et al. evaluated 164 pediatric patients with papillary thyroid carcinoma and reported a validation AUC of 0.832 for predicting extrathyroidal extension, outperforming routine sonographic assessment [79]. This is clinically relevant because extrathyroidal extension affects operative planning. However, class imbalance, manual segmentation, and single-center validation limit immediate clinical use.

Wei et al. reported high performance for benign-malignant differentiation of subpleural pulmonary lesions in 609 patients, with validation AUC of 0.924 [80]. Although not exclusively pediatric, this study illustrates the potential of combining radiomics, deep learning features, and clinical variables. Its limitations also highlight key barriers for ultrasound radiomics: retrospective design, class imbalance, grayscale-only imaging, and use of non-DICOM images.

#### 5.4.3. Critical Summary

Oncologic ultrasound radiomics should be viewed as an adjunctive risk-stratification tool rather than a stand-alone diagnostic method. Future studies must address small sample size, class imbalance, and external validation. The most valuable models will likely be multimodal, combining ultrasound radiomics with clinical features, pathology, molecular markers, and outcomes.

### 5.5. Emerging Applications

Lung and musculoskeletal applications illustrate the expanding scope of pediatric ultrasound radiomics, but these areas are heterogeneous and less mature. Many studies overlap with quantitative ultrasound rather than conventional radiomics, so interpretation requires caution.

#### 5.5.1. Lung Ultrasound Radiomics

Lin et al. evaluated 150 neonatal lung disease cases and reported strong performance for neonatal respiratory distress syndrome classification, with a validation AUC of 0.951 [81]. This suggests that radiomics may reduce operator dependence in neonatal lung ultrasound.

Cai et al. evaluated 301 pediatric patients with acute dyspnea and reported an AUC of 0.976 for an integrated radiomic–clinical model for acute heart failure diagnosis [82]. Although promising, the high performance should be interpreted cautiously because of overfitting concerns and the need for comparison with expert clinical assessment.

Mohamed, Sultan, Venkatakrishna, and colleagues explored AI-assisted quantitative lung ultrasound analysis for pediatric pneumonia-related findings in a small pilot cohort [83]. This remains preliminary, but it highlights a clinically relevant direction: semi-automated quantification of consolidation, pleural abnormalities, and B-lines.

#### 5.5.2. Musculoskeletal and Muscle Applications

Quantitative muscle ultrasound studies in Duchenne muscular dystrophy show that grayscale intensity and backscatter features can distinguish diseased from healthy muscle and correlate with functional status [84,85,86]. These findings support the broader value of ultrasound-derived quantitative biomarkers, although not all studies represent conventional radiomics.

Scatteromics and hip ultrasound radiomics further extend this concept. Advanced scatter-based analysis has shown potential for distinguishing ambulatory decline in Duchenne muscular dystrophy [87], while developmental dysplasia of the hip radiomics achieved validation AUC of 0.91 for detecting femoral head microstructural changes [88]. These findings are promising, but the clinical role of these methods remains to be defined.

#### 5.5.3. Critical Summary

Emerging lung and musculoskeletal applications suggest that radiomics may expand beyond traditional organ-based imaging into bedside functional and structural monitoring. However, the evidence remains early, heterogeneous, and often single-center. Future studies should clarify whether these quantitative approaches provide incremental value beyond expert interpretation, clinical scores, and established quantitative ultrasound measures.

**Table 1:** Summary of pediatric ultrasound radiomics studies across organ systems, including study population, clinical application, segmentation approach, radiomic feature-extraction methodology, model performance, major limitations, and level of evidence.

	Sample Size and Subject Age	Primary Disease/Purpose/Clinical Task	Segmentation Method/Radiomics Pipeline	Radiomic Features & Models Used/Validation (Model Algorithm, Logistic Regression, Training/Testing/Division)	Model Used/Performance	Limitations (Validation, Single Center, Sample Size, Study Design)	Evidence Level (Oxford Center for Evidence-Based Medicine)
**Brain**							
(Narchi et al. 2013 [60])	20 preterm neonates (Mean GA 28.5 ± 1.9 weeks)	White Matter Injury (WMI)-Cystic Periventricular Leukomalacia (PVL) Purpose: Test whether texture analysis of PVE on initial cranial US can differentiate transient echogenicities (which resolve) from those that develop into cystic PVL	Manual ROI segmentation-Software: MaZda v4.5 (B11) Feature extraction–305 features per image	Co-occurrence matrix (COM), run-length matrix (RLM), and the gradient matrix (Gr) Top 10 features selected by Fisher (F)-coefficient	Classifier: Linear discriminant analysis (LDA) → two most discriminant features (MDF1, MDF2) Validation: Leave-one-out cross-validation	- Lack of correlation to PVL risk factors - Lack of long-term follow-up - No standardization of US settings - Small sample size - No multivariate analysis - Single center - Retrospective observational case-control study - Risk of over-fitting (over-conformity) due to small sample size relative to the dimensionality of radiomic feature space	Level 3b
(You et al. 2015 [61])	33 preterm neonates (Mean GA 28.45 ± 2.7 weeks)	WMI–Diffuse cystic or cavitary periventricular changes Purpose: Quantitatively analyze GLCM texture features on cranial sonography and correlate with WMI severity graded by MRI (normal vs. mild vs. severe)	Manual ROI segmentation Software ImageJ v1.44 and In-house C++ software (Medical Imaging Solution for Segmentation and Texture Analysis) MRI grading by 2 blinded board-certified pediatric radiologists 48 features per image	Angular second moment (ASM), inverse differential moment (IDM), contrast, and entropy Statistical analysis: Kruskal-Wallis test, post-hoc Mann-Whitney U (Bonferroni-corrected α = 0.017) ROC curve analysis for discrimination No ML model; statistical comparison with MRI-based severity grading	Custom GLCM pipeline	- Retrospective study - Single center - Different scanner settings - Interval between US and MRI - Small sample size - No image normalization/postprocessing - Could not discriminate mild WMI from normal - No neurodevelopmental outcome correlation - Risk of over-fitting (over-conformity) due to small sample size relative to the dimensionality of radiomic feature space	Level 3b
(Zhu et al. 2023 [62])	158 preterm neonates (GA < 37 weeks, age ≤ 7 days)	WMI-Cystic PVL Purpose: Develop an ultrasound radiomics diagnostic system combining traditional radiomics and multi-task deep learning (MTDL-Net) for automated WM segmentation and WMI risk prediction from cranial US images	Manual segmentation by radiologists (≥10 years experience) as gold standard ROIs Automatic segmentation: SDL-Net (U-Net) and MTDL-Net (Mask R-CNN) Software: PyTorch, MATLAB for preprocessing followed by feature extraction–350 features per image	Histogram statistics, GLCM, GLRLM- based features, gray-level size zone matrix (GLSZM) based features, neighboring gray tone difference matrix (NGTDM) based features, and four wavelet-transformed modes Feature selection: Sparse representation-based classification (SRC) → top 52 features	Classifier: SVM-based C-SVC Deep learning: MTDL-Net (Mask R-CNN) for simultaneous segmentation + classification Fusion: deep learning features + manual radiomics features Validation: 70:30 train-test split; 5-fold cross-validation for MTDL-Net (avg AUC 0.843)	Retrospective study Single center Small sample size with class imbalance (32 WMI vs. 126 normal) No external validation Static image-based analysis (not video/cine) PVL not separately categorized Limited to anterior fontanelle coronal plane No neurodevelopmental outcome correlation - Risk of over-fitting (over-conformity) due to small sample size relative to the dimensionality of radiomic feature space	Level 3b
(Zhu et al. 2024 [63])	267 premature infants (GA 26–36 weeks, mean GA 32.55 ± 2.55 weeks)	White Matter Injury (WMI) Purpose: Develop and evaluate a multiplanar radiomics model from CUS to predict WMI, dynamically monitor WMI recovery at 2 and 4 weeks, and explore correlation with neurodevelopment	Manual ROI segmentation followed by voxel intensity fixed at 25, voxel size resampled 1 × 1 mm, then feature extraction via Pyradiomics 3.0.1 software	Shape, first-order, GLCM, GLRLM, GLSZM, GLDM, NGTDM Feature selection: Spearman correlation + LASSO with 5-fold cross-validation → 58 features	Least absolute shrinkage and selection operator (LASSO) algorithm	Retrospective study Small sample size Limited vendor/probe types (2 machines) No independent external validation Does not classify WMI severity (mild/moderate/severe) Manual ROI segmentation (inefficient) Low sensitivity for microcystic (<1mm) and non-cystic diffuse WMI Single center - Risk of over-fitting (over-conformity) due to small sample size relative to the dimensionality of radiomic feature space	Level 3b
(Jung et al. 2019 [64])	N = 20 very preterm infants (GA 25–33 weeks)	WMI-PVL Purpose: Compare texture parameters of serial CUS images between PVL and normal PVE groups; evaluate early predictive value of texture analysis for PVL within 2–3 weeks of life	Manual ROI segmentation: Software: MaZda v4.5 (Technical University of Lodz) Normalization: histogram remapping within ±3 SD of mean Blinded neuroradiologist (4 years CUS experience)	Features: 308 texture features + variance-to-mean ratio (VMR); first-order histogram (variance, Perc.99%, MaxNorm), co-occurrence matrix, run-length, gradient features Statistical tests: Wilcoxon signed-rank, Mann-Whitney U ROC analysis for discrimination No ML model; statistical comparison approach No train/test split	Key metric: R21 of VMR (ratio of 2nd to 1st CUS values)	Retrospective study design Very small sample size Single center PVL diagnosed by CUS or MRI (not all had MRI)—possible false negatives in normal PVE group PVL severity not stratified Scanner settings not standardized (though same machine/operator used) - Risk of over-fitting (over-conformity) due to small sample size relative to the dimensionality of radiomic feature space	Level 3b
(Laccetta et al. 2023 [65])	N = 46 preterm infants (GA < 32 weeks)	Non-cystic WMI Purpose: Evaluate quantitative CUS echogenicity of periventricular WM (pixel brightness intensity) as a predictor of middle-term neurodevelopment (Bayley-III at 12 mo CA)	Manual segmentation: Software: QLAB13 (Philips, Amsterdam, The Netherlands) Metric: Relative echogenicity (RECP) = mPBI_WMmax/mPBI_CPmax	Feature: Pixel brightness intensity (PBI)— first-order intensity ratio (RECP) Statistical models: Pearson correlation, multivariate linear regression with covariates (GA, arterial pH, PN duration, hospital stay, ROP, hsPDA) No train/test split (single cohort analysis)	No ML classifier; correlational/regression approach	Small sample size Single center Bayley-III at 12 months may be early for cognitive/language assessment Operators aware of clinical history (though blinded to study aims) Acoustic window changes with age No MRI correlation - Risk of over-fitting (over-conformity) due to small sample size relative to the dimensionality of radiomic feature space	Level 2b
(Zimina et al. 2026 [66])	N = 89 full-term newborns (GA > 37 weeks)	Diabetic fetopathy in newborns born to mothers with gestational diabetes mellitus Purpose: Investigate feasibility of radiomic analysis of brain US images to detect brain changes associated with diabetic fetopathy not visible on standard neurosonography	Manual segmentation Software: PyRadiomics (Python 3.9, Visual Studio 2022) 1395 features extracted per image	Feature classes: first-order, shape, GLCM, GLRLM, GLSZM, GLDM, NGTDM Feature selection: Shapiro-Wilk → *t*-test/Mann-Whitney U.; Spearman correlation filtering (|r| < 0.7) Validation: 80/20 train-test split with stratification; repeated stratified 5-fold cross-validation StandardScaler normalization	Model: Decision tree classifiers (scikit-learn) with automatic hyperparameter tuning	Small sample size Single center Retrospective design No external validation cohort No neurodevelopmental outcome correlation Overfitting concern (Model 4 test AUC 0.85 vs. CV AUC 0.65) Color-to-grayscale conversion may introduce artifacts - Risk of over-fitting (over-conformity) due to small sample size relative to the dimensionality of radiomic feature space	Level 3b
(Sultan et al. 2025 [67])	N = 33 full-term newborns (≥37 weeks GA)	HIV-exposed uninfected (HEU) vs. HIV-unexposed (HU) full-term newborns Purpose: Explore brain ultrasound radiomics (texture analysis) as early neurodevelopmental biomarker comparing by in utero HIV exposure status	Manual ROI segmentation Software: MaZda v4.6 (Technical University of Lodz) Blinded expert (10 years experience) reviewed and excluded artifact-affected images	Features: 12 features including; First-order statistics (heterogeneity), GLCM (entropy, correlation), run-length matrix (RLNonUni, GLevNonU) Validation: ROC analysis with AUC, sensitivity, specificity No formal train/test split described; single cohort analysis	Model: Logistic regression combining 5 features	Small sample size Single site Cross-sectional (no longitudinal neurodevelopmental follow-up) Homogeneous ART exposure (all dolutegravir-based) Images acquired by study nurses (point-of-care US) 14 of 47 studies excluded for image quality No external validation - Risk of over-fitting (over-conformity) due to small sample size relative to the dimensionality of radiomic feature space	Level 2b
**Liver**							
(Das et al. 2021 [71])	181 pediatric participants in total. Model development dataset contained 132 subjects: 93 normal subjects (484 ROIs) and 39 NAFLD subjects (260 ROIs) used to develop the ML model Age: 6–18 years old	Pediatric Non-alcoholic fatty liver disease (NAFLD) Purpose: Develop a machine learning (ML) based classification model capable of identifying NAFLD from healthy liver tissue using ultrasound texture analysis.	Manual ROI segmentation of 25 × 25 pixel rectangles per image. Texture extraction was performed with ImageJ and MaZda software.	28 texture features including histogram, co-occurrence matrix (GLCM), run-length matrix, gradient, autoregressive, and Haar wavelet features	Best models: Support Vector Machine, multi-layered perceptron neural net, and extreme gradient boost Testing AUROC 0.95 (95% CI 0.93–0.97). Internal validation AUROC 0.969. External validation AUROC 0.92 (95% CI 0.91–0.94). Outperformed HRI-only (AUROC 0.81), HEAI-only (0.75), and combined HRI + HEAI (0.82).	- Single-center study Relatively small number of NAFLD - Retrospective secondary analysis of a prospective cohort - Manual ROI placement	Level 3b
**Renal**							
(De Leon-Benedetti et al. 2025 [25])	31 pediatric subjects (60 kidney units): 8 AKI (median age 3.5 years [IQR: 0–11.5]); 14 CKD (median 3.5 years [IQR: 0–6.8]); 9 healthy controls (median 15.5 years [IQR: 12.8–21])	To determine whether grayscale US radiomics can differentiate AKI vs. CKD vs. healthy kidneys. Purpose: can US radiomics quantify ‘medical renal disease’ beyond subjective echogenicity	Manual ROI segmentation of renal parenchyma on sagittal long-axis B-mode US images by trained researchers with pediatric nephrologist and radiologist review Software: MaZda v4.6 (Technical University of Lodz) Gray level normalization before feature extraction, within each ROI, to rescale pixel intensities to standardized range of 0–255	Feature extraction (124 total) using PyFeats v1.0.11: GLCM, GLDS, NGTDM, SFM, LTE, FDTA, GLRLM, FPS, GLSZM, HOS, LBP and wavelet packet decomposition Model: principal component analysis–top 10 features selected for modeling. Features used to evaluate 4 ML models: random forest, SVM with radial basis function kernel, logistic regression, and XGBoost Validation: 5-fold cross validation only	Best model: XGBoost, accuracy 0.90, macro F1 0.90 Other models: SVM accuracy 0.90, macro F1 0.88; random forest accuracy 0.88, macro F1 0.89; logistic regression accuracy 0.88, macro F1 0.88	Retrospective pilot study, very small cohort, single-center, no external validation, age not matched between controls and disease groups; heterogeneous US machines, transduces and settings; manual segmentation Risk of over-fitting (over-conformity) due to small sample size relative to the dimensionality of radiomic feature space	Level 3b
(Kou et al. 2024 [74])	313 biopsy confirmed pediatric glomerulonephropathy cases: 127 IgA nephropathy, 83 minimal change disease, 103 henoch-schonlein purpura nephritis 469 renal US images used total Age not specified, but pediatric cases	To noninvasively differentiate biopsy-confirmed pediatric GN subtypes (IgAN vs. MCD vs. HSPN)	Manual ROI segmentation of renal parenchymal trasnverse B-mode US images acquired immediately prior to biopsy using LabelMe cropped to standardized 512 × 512 pixels. Then a U-Net segmentation model was trained on manually segmented images.	1422 features extracted with PyRadiomics: first order features, shape, GLCM, GLSZM, GLRLM, NGTDM and GLDM. Random 8:2 split into training and validation sets. ANOVA, LASSO regression and k-fold cross-validation to determine optimal subset of features. Then RF classification model used to evaluate selected features. No external validation	Final classification model: Random Forest with 37 selected radiomic features Validation AUCs: IgAN (0.94); MCD (0.91); HSPN (0.98)	Single-center study; only 3 subtypes of GN included with no healthy controls; no external validation; age/sex/clinical characteristics not reported; possible data leakage if image-level not patient-level splitting occured Risk of overfitting (over-conformity) due to 1422 initial features (large original feature space) extracted relative to only 313 cases All images acquired on one US system with same probe/settings improves standardization but limits results generalizability	Level 3b
(Chen et al. 2024 [75])	440 biopsy-confirmed HSPN patients. Grouped (n = 100) ISKDC I-II (no cresecents) vs. (n = 340) ISKDC III-V (crescentic) Training cohort 308, validation 132 (7:3 split). Median training cohort age 9 years (IQR: 6–11), validation cohort median age 8 years (IQR: 5–11)	HSPN classification using renal US radiomics Purpose: to predict crescentic vs. non-crescentic using biopsy proven ISKDC grading through a noninvasive surrogate	Manual ROI segmentation of renal cortex and medulla (excluding collecting system/hilum) using largest long-axis US image of right kidney in 3D slicer. Segmentations performed by a sonographer and reviewed by a second sonographer. No automated segmentation or deep learning pipeline	105 radiomic features extracted with Pyradiomics: first-order, shape, GLCM, GLSZM, GLRLM, NGTDM and GLDM Feature selection with spearman correlation filtering (if > 0.9, feature removed) then LASSO regression with 5-fold cross-validation: 14 features selected 14 features (3 First order, 4 shape [2D], 7 texture) used to build 3 ML models: logistic regression, k-nearest neighbor, and SVM Internal validation using the n = 132 validation cohort	Best performing model: SVM-Training AUC 0.910, validation AUC 0.870 (95% CI 0.795–0.944). Validation sensitivity 0.706, specificity 0.950, accuracy 0.761, F1 score 0.821 KNN validation AUC 0.810 (95% CI 0.712–0.909) LR validation AUC 0.751 (95% CI 0.625–0.878)	Retrospective, single-center, internal validation only, manual segmentation, feature reproducibility/interobserver ICCs not reported	Level 3b
(Sloan et al. 2023 [76])	592 US images 90 patients (ages 0–8 years) with clinical diagnosis of hydronephrosis based on Society for Fetal Urology (SFU) grading system: categorized to low-grade (SFU I-II) and high-grade (SFU III-IV). 74 high-grade kidneys (145 images) and 227 low-grade kidneys (447 images) were included	Pediatric hydronephrosis severity classification based on US Purpose: To develop a ML algorithm based on radiomic texture features to differentiate SFU-defined low-grade from high-grade pediatric hydronephrosis	Each kidney manually outlined by urology resident with pediatric urologist expert review (5% required re-contouring) No automated segmentation	25 chosen radiomic features extracted: 14 GLCM-derived (contrast, entropy, homogeneity, IMC1/2, correlation etc.), grayscale statistics, and morphologic features (circularity, compactness, effective diameter, depth-to-width ratio) 5-fold cross-validation (constant low-grade to high-grade ratio in each fold & patients/kidneys kept within folds to prevent data leakage). Stepwise linear discriminant using Wilks’ lambda as the feature selection criterion during training folds. Linear SVM used as final classifier	SVM performance analyzed by AUC of ROC curve output. Mann-Kendall test used to determine if positive correlation between SVM output and hydronephrosis grade. By kidney unit, AUC 0.86 (95% CI 0.81–0.92), sensitivity 75.7%, specificity 86.3%, accuracy 83.7%, PPV 64.4%, NPV 91.6%, F1-score 69.6%. Significant positive trend between model ouput and increasing SFU grade (*p* < 0.001)	Retrospective, single-center pilot study; relatively small patient cohort; excluded many structural abnormalities; manual segmentation may introduce observer variability; possible image-selection bias; standardization of image acquisition unclear from text (“main ultrasound system”) Lower risk of over-fitting (over-conformity) as only 25 features extracted, reduced data leakage (patient kept within a validation fold), and cross-validation by kidney (not image)	Level 3b
**Oncology**							
(Zhu et al. 2025 [78])	73 pediatric patients in total. 25 cases of ganglioneuroma (GN), 12 cases of ganglioneuroblastoma intermixed (GNBi), 2 cases of ganglioneuroblastoma nodular (GNBn), and 34 cases of neuroblastoma (NB). Mean age 45.5 ± 39.8 months (range 0.4–180 months)	Peripheral neuroblastic tumors Purpose: Construct and select a better model for prognostic subsets of pediatric neuroblastic tumors using US.	Images of the largest sections of tumors were chosen for feature extraction. Radiomics feature extraction using Pyradiomics.	1674 radiomics features were extracted. 324 first-order statistical features and 1350 textural features including GLCM, GLDM, GLRLM, GLSZM and NGTDM. Filters applied include Laplacian of Gaussian (LOG), wavelet with transform, square, square root, logarithm, exponential and gradient. LASSO regression was used for combined models.	Best model: Radiomics model surpassed the combined model at differentiating prognostic subsets of pNTs (AUC 0.918), despite the combined model having the highest AUC (0.941) with *p* < 0.05 Radiomics models used included RF, KNN, LR, SVM, and xgboost.	- retrospective data - limited sample size - Mono-center cohort study - imbalance between favorable and unfavorable histology groups	Level 3b
(Wei et al. 2026 [80])	609 patients were included; 422 diagnosed with malignant conditions, and 187 with benign tumors. 487 cases were used for training, and 122 cases were used for validation. Mean age was not specified.	Subpleural pulmonary lesions (SPLs). Purpose: develop a clinical deep learning model (CDLR) for differential diagnosis of benign and malignant SPLs using US.	Images were exported in JPG format and uniformly converted to NIfTI format using SimpleITK library before being imported into Insight Segmentation and Registration Toolkit SNAP (ITK-SNAP) software. Manual segmentation and ROI delineation. Radiomics and deep transfer learning (DTL) features were extracted using Pyradiomics.	1561 radiomics features were extracted, including include shape, first-order, and texture features. 128 deep transfer learning features were identified.	DTL and DLR models demonstrated superior value to the RAD model. Clinical, RAD and DTL features were integrated through a SVM algorithm model to construct the CDLR model.	- retrospective cohort study - single center study - only grayscale ultrasound was used - use of JPG rather than DICOM images - class imbalance between malignant and benign lesions	Level 3b
(Li et al. 2023 [79])	164 pediatric patients in total; 103 with pathologically identified ETE, and 61 non-ETE. 115 were used for training, and 49 were used for validation. Mean age was 14.60 ± 3.52 years.	Papillary thyroid carcinoma Purpose: explore extrathyroidal extension (ETE) in children and adolescents.	ROI delineation was performed layer by layer along the edge of the tumor contour using ITK-SNAP software. ETE diagnosis according to AKCC standards.	1421 image features were extracted using Pyradiomics. Features were divided into 4 categories: shape, first-order statistics, texture, and higher-order statistical features. 217 features with correlation coefficient >0.90. 16 radiomics features were chosen using LASSO.	Best model: radiomics-random forest (RF) model with AUC of 0.999 in the training set, and LightGBM model with AUC of 0.832. Models used included KNN, SVM, RF, and LightGBM.	- retrospective cohort study - single center cohort - small sample size - Imbalance between ETE and non-ETE cases - Did not compare dimensionality reduction algorithms - tumor boundaries were ill-defined in some instances - Only greyscale ultrasound images were used	Level 3b
**Emerging Areas**							
(Lin et al. 2024 [81])	150 consecutive cases of neonatal lung disease in one NICU: NRDS (n = 60), neonatal pneumonia (n = 30), MAS (n = 30), TTN (n = 30) 8:2 split --> Training cohort (n = 120), median age 263 days (IQR 216–277); validation cohort (n = 30), median age 264 days (IQR 228–277)	In patients with neonatal respiratory distress syndrome (NRDS), investigation of lung ultrasound radiomics Purpose: to develop an operator-independent US radiomics model to improve diagnostic consistency in neonatal lung US for NRDS	All US images acquired on one US system (GE LOGIQ P6) with standardized probe (9–12 MHz linear) and settings Lesion ROIs manually segmented by two senior physicians specializing in neonatal lung US in ITK-SNAP 3.8.0 Two representative images saved for each patient in DICOM format	107 image features extracted using PyRadiomics including GLCM (n = 24), first-order (n = 18), GLRLM (n = 16), GLSZM (n = 16), GLDM (n = 14), shape (n = 14), NGTDM (n = 5). Multiple features combined if spearman correlation > 0.9, greedy recursive strategy used to filter irrelevant features. Remaining figures underwent LASSO regression with 10-fold cross-validation Final model used 22 non-zero features	ML models, RF, SVM, MLO, KNN, and logistic regression employed with validation by a temporally separate internal cohort Best performing model: Random Forest. Validation cohort AUC 0.951, sensitivity 95,83%, specificity 94.44%, accuracy 95.0%, PPV 92.0%, NPV 97.14%. RF model performance was statistically comparable with experienced physicians (AUC 0.99 vs. 0.98) and significantly better than junior physicians (AUC 0.85)	Single-center retrospective study; relatively small cohort; no external validation; single US system standardized image acquisition; manual ROI segmentation introduces observer variability; potential for model dependence on institution-specific acquisition settings; possible image-level data leakage concerns [2 images per patient’ not fully clarified in methods Risk of over-fitting (over-conformity): small dataset relative to classification complexity; due to single-vendor acquisition—study generalizability limited; possible image-selection bias for model training has potential to make ML model classification easier than in real-world with greater clinical variability	Level 3b
(Cai et al. 2026 [82])	301 patients were included. 210 were used for training, and 91 were used for testing. Age range was 1–18 years old.	Acute heart failure (AHF) Purpose: develop an integrated machine learning model based on lung US radiomics and clinical data to diganogs AHF in patients with acute dyspnea.	Standardized 6-zone lung US images were used. Images in DICOM format were imported to 3D Slicer software. ROI delineation was manually performed.	159 features were used in total: 107 radiomics features were extracted using Pyradiomics and combined with 52 clinical features. Features included shape, first-order, and texture features such as GLCM, GLRLM, GLSZM, GLDM and NGTDM. Synthetic Minority Oversampling Technique (SMOTE) was applied to account for the mild class imbalance.	Best model: the integrated RF model achieved optimal performance with an AUC of 0.976 (95% CI: 0.950–0.994), particularly GLRLM texture features. 3 RF models were developed: clinical-only, radiomics-only, and an integrated model.	- retrospective study - single-center cohort study - feature-to-event ratio was 1:0.93, which is below the recommended 10:1, posing a risk of overfitting - experts were blinded to radiomics features, leading to potential artificially inflated performance of the model. - model was not compared to physicians’ diagnostic performance - model used 6-zone protocol rather than the internationally recommended 8- or 12-zone standard protocol.	Level 3b
(Mohamed et al. 2026 [83])	10 pediatric cases in total. 5 cases with pneumonia findings, and 5 cases with normal findings. Age range was 3–32 months.	Pediatric Pneumonia-related US findings Purpose: evaluate semi-automated, AI-assisted system at identifying clinically relevant lung abnormalities on US.	Automated segmentation algorithm developed by the authors that targets key pathological features such as consolidation, pleural line abnormalities, and B-lines via region growing, selecting pixels with similar greyscale intensity values (±10 gray levels). Minor manual correction was performed.	Quantitative features extracted include tissue thickness, margin irregularity, and variation in echogenicity. Custom-developed software in IDL based on computerized analysis of echogenicity variation, margin irregularity, and structural continuity.	Accuracy in detecting consolidation, B-lines and pleural lines.	- small sample size - validation consists of simple visual confirmation	Level 3b
(Wijntjes et al. 2022) [86])	Predominantly adult but pediatric subset included 994 patients: pediatric subgroup limited to <5 years (n = 50), 5% & 6–18 years (n = 112), 11.3%	In patients with neuromuscular disease (myopathies, neurogenic, NMJ disorders, and non-muscle NMDs), comparison of visual muscle ultrasound assessment (Heckmatt grading) and quantitative muscle ultrasound echogenicity z-score analysis Purpose: to determine the correlation between qualitative and QMUS	No traditional radiomic feature extraction. Manually segmented ROIs representing maximal muscle area with computed echogenicity quantification scores	No ML classifier or advanced radiomic texture pipeline was developed	Not Applicable	Not applicable	
(Shklyar et al. (2015) [84])	25 boys with Duchenne muscular dystrophy (DMD) and 25 healthy controls Age range: 2–14 years	Duchenne muscular dystrophy Purpose: Compare grayscale level (GSL) and quantitative backscatter analysis (QBA) for differentiating DMD muscle from healthy muscle and examine relationships with age and functional status.	No conventional radiomics pipeline. Quantitative muscle US measurements were obtained from 6 unilateral muscles. GSL derived from standard grayscale US images; QBA derived from raw backscatter signal data requiring specialized processing.	Quantitative US intensity/scattering measures: GSL and QBA. Reliability assessed with intraclass correlation coefficients (ICCs). Associations evaluated with age and functional status (North Star Ambulatory Assessment). No ML classifier, train/test split, or radiomics feature-selection pipeline.	Both GSL and QBA were highly reliable (ICC > = 0.87) and differentiated DMD from controls. Superficial muscle measurements increased with age and worsening functional status, suggesting that anatomic sampling strategy affects diagnostic sensitivity. GSL may be more feasible for routine use because it can be extracted from standard US images.	Small single-center case-control cohort; not a conventional grayscale radiomics study; QBA requires raw signal data and specialized processing; no external validation; no ML model; potential dependence on acquisition settings and muscle depth/sampling location.	Level 3b
(Chuang et al. 2025 [87])	47 boys with Duchenne muscular dystrophy	Duchenne muscular dystrophy Purpose: Introduce quantitative US-based scatteromics to distinguish early vs. late ambulatory decline in DMD using gastrocnemius ultrasound envelope-statistics parametric imaging.	Not conventional grayscale radiomics. Gastrocnemius ultrasound envelope-statistics parametric maps were generated. Maps were based on Nakagami, homodyned K, and entropy parameters; first-order features were extracted from parametric images.	Scatteromics features derived from Nakagami, homodyned K, and entropy parametric maps. Classifiers: support vector machine (SVM), random forest (RF), and linear discriminant analysis (LDA). Validation approach not fully detailed in the review table source text.	Full scatteromics models achieved high average AUROCs: SVM 0.97, RF 0.98, and LDA 0.83. Findings support ultrasound-derived scattering/texture features as objective biomarkers of pediatric muscle disease severity.	Small cohort; disease-specific DMD population; not a conventional grayscale radiomics workflow; requires quantitative US envelope-statistics processing and parametric map generation; external validation and broader multicenter testing are needed; potential risk of over-fitting relative to sample size and feature/model complexity.	Level 3b
(Hao et al. 2025 [88])	125 infants total: 59 with developmental dysplasia of the hip (DDH) and 66 healthy controls	Developmental dysplasia of the hip Purpose: Evaluate whether hip US radiomics can identify microstructural femoral head changes in DDH and provide a quantitative adjunct to conventional Graf-based US assessment.	Femoral head ROIs manually segmented on hip US images using 3D Slicer, excluding the ossification center. Retrospective study design.	92 radiomic features extracted, including first-order, GLCM, gray-level dependence matrix (GLDM), and gray-level size zone matrix (GLSZM) features. Feature selection: maximum relevance minimum redundancy (mRMR) and LASSO regression. Final model: 9 selected radiomic features.	69 features differed significantly between DDH and controls. Nine-feature radiomics model achieved validation AUC 0.91. Suggests US radiomics may capture subtle femoral head tissue alterations in DDH.	Retrospective study; modest sample size; likely single-center cohort; manual segmentation; no multicenter external validation described; model requires prospective validation and assessment of reproducibility across scanners/operators; possible risk of over-fitting given initial feature number relative to cohort size.	Level 3b

## 6. Future Directions Toward Clinical Translation and Real-World Implementation

Despite growing interest in ultrasound radiomics, several critical steps remain before these approaches can be translated into routine clinical care. Future progress will depend not only on technical refinement, but also on demonstrating that ultrasound radiomics can provide actionable information at the point of care. For clinical implementation, radiomic tools must be reliable across scanners and operators, interpretable to clinicians, integrated into existing ultrasound workflows, and supported by evidence that they improve diagnostic confidence, risk stratification, treatment monitoring, or patient outcomes. Therefore, the path forward is inherently multidisciplinary, requiring collaboration among imaging scientists, pediatric radiologists, sonographers, engineers, informatics teams, industry partners, and regulatory bodies.

### 6.1. Standardization as the Foundation for Clinical Use

A major priority for clinical translation is the standardization of ultrasound radiomics methodology. Unlike more standardized cross-sectional imaging modalities, ultrasound is highly operator-dependent and sensitive to acquisition parameters, including transducer selection, frequency, gain, depth, focal zone placement, transducer pressure, and vendor-specific image processing. These sources of variability can substantially influence radiomic feature stability and may limit reproducibility across institutions and platforms.

Accordingly, future efforts should focus on establishing clinically practical acquisition protocols that can be implemented by sonographers in routine practice. These protocols should define not only scanner settings and image planes, but also minimum image-quality requirements, acceptable artifact thresholds, and standardized documentation of acquisition parameters. In parallel, preprocessing and feature-extraction pipelines must be harmonized so that radiomic outputs are reproducible across scanners, vendors, operators, and clinical environments. Reproducibility studies will be essential to identify robust features that remain stable under real-world imaging conditions and can therefore serve as reliable ultrasound-derived imaging biomarkers.

### 6.2. Multicenter and Multi-Vendor Validation

Beyond technical standardization, multicenter and multi-vendor validation is essential to determine whether ultrasound radiomic signatures are generalizable. Much of the current literature remains based on relatively small, single-center cohorts, often with limited external validation. While these studies are valuable for proof of concept, they are insufficient to establish broadly applicable biomarkers.

Large, diverse datasets collected across institutions, patient populations, disease subtypes, age groups, and imaging platforms are needed to test the robustness and transportability of proposed models. This is particularly important in pediatric imaging, where disease rarity, developmental variation, and differences in scanning practice can limit generalizability. Multicenter validation should also evaluate whether radiomics provides incremental value beyond conventional ultrasound interpretation, Doppler assessment, contrast-enhanced ultrasound, laboratory markers, pathology, MRI, or clinical outcomes. Demonstrating this added value will be essential for clinical adoption.

### 6.3. Integration into the Ultrasound Workflow

For ultrasound radiomics to become clinically meaningful, it must be integrated into the workflow in a way that is efficient, interpretable, and minimally disruptive. Radiomics should not require complex offline analysis that delays reporting or depends on specialized research personnel. Instead, future systems should aim for automated or semiautomated workflows that include image-quality assessment, region-of-interest detection, segmentation, feature extraction, and quantitative output generation within or alongside existing ultrasound platforms and PACS/reporting environments.

Near real-time analysis may allow for radiomic outputs to be generated during image acquisition or interpretation. For example, radiomics could provide immediate feedback on whether image quality is sufficient, whether the region of interest has been appropriately captured, or whether quantitative texture features fall outside an expected range. Such integration would make radiomics more practical for clinical use and could reduce operator burden while improving consistency across examinations.

### 6.4. Clinically Actionable Decision Support

The greatest clinical value of ultrasound radiomics will likely come from decision-support tools rather than isolated feature values. Radiomic outputs should be translated into clinically understandable information, such as probability scores, risk categories, longitudinal change metrics, or alerts for abnormal tissue patterns. These outputs should be presented in a way that supports, rather than replaces, expert radiologist interpretation.

In practice, ultrasound radiomics may be most useful when combined with grayscale findings, Doppler features, contrast-enhanced ultrasound metrics, laboratory data, pathology results, and clinical context. Such integrated models could assist with lesion characterization, disease staging, treatment response assessment, risk stratification, and follow-up planning. Importantly, these tools should be designed around specific clinical questions, such as distinguishing acute from chronic kidney injury, identifying early brain maturation differences, monitoring liver fibrosis, assessing tumor risk, or quantifying muscle and lung disease progression.

### 6.5. Application-Specific Implementation Pathways

Future work in pediatric ultrasound radiomics should move beyond broad technical feasibility and focus on application-specific pathways to clinical relevance. In neonatal brain imaging, longitudinal and multiplanar radiomics approaches may be particularly valuable when linked to neurodevelopmental outcomes, as serial textural change may provide more clinically meaningful information than single-timepoint classification alone.

In liver imaging, future studies should prioritize pediatric-specific validation and evaluate how radiomics can complement elastography, Doppler, contrast-enhanced ultrasound, laboratory markers, and clinical staging. In renal applications, radiomics should be developed not only to differentiate disease categories but also to support treatment monitoring, prediction of functional decline, and correlation with pathology or clinical outcomes. In oncologic imaging, clinically useful models should focus on risk stratification, biopsy guidance, treatment response, and follow-up decisions. Emerging lung and muscle applications further suggest that ultrasound radiomics may support functional and interpretable biomarkers, including pleural-line analysis, disease monitoring, and AI-guided automated assessment.

Across these domains, successful translation will require models that answer focused clinical questions, produce interpretable outputs, and demonstrate measurable value beyond standard ultrasound interpretation.

### 6.6. AI-Enabled Ultrasound Radiomics Platforms

Future development of ultrasound radiomics may involve closer integration with artificial intelligence, including machine learning and deep learning methods. Rather than serving only as isolated quantitative descriptors, radiomic features could potentially be incorporated into broader multimodal models that combine handcrafted imaging features, learned image representations, and relevant clinical data.

In ultrasound, AI-based methods may eventually assist with selected components of the radiomics workflow, such as acquisition guidance, image-quality assessment, region-of-interest identification, segmentation, feature extraction, or predictive modeling. However, these applications remain largely investigational in pediatric ultrasound radiomics, and their clinical utility has not yet been established in routine practice. Therefore, AI-enabled radiomics platforms should be viewed as a potential future direction rather than a currently validated clinical solution.

Any future implementation will require transparent model development, rigorous validation, clear communication of uncertainty, and continued human oversight. For radiologists and clinicians to trust these tools, outputs must be interpretable, reproducible, and clinically relevant. Importantly, AI-based radiomics should support, rather than replace, expert ultrasound interpretation.

### 6.7. Regulatory Approval, Reporting, and Clinical Adoption

Successful translation will also depend on addressing regulatory and implementation challenges. Before clinical deployment, ultrasound radiomics tools must undergo rigorous technical and clinical validation to demonstrate reliability, reproducibility, safety, and clinical utility. Regulatory approval pathways will require evidence that these systems perform consistently across intended use settings, including different scanners, operators, institutions, and patient populations.

Equally important is physician acceptance. Even technically strong tools are unlikely to be adopted unless they are transparent, interpretable, easy to use, and clearly beneficial to patient care. Integration into radiology reporting systems will therefore be crucial. Rather than presenting large numbers of abstract features, future reports should provide concise, clinically meaningful summaries, such as risk estimates, quantitative change over time, or decision-support statements. Clinical adoption will ultimately depend not only on algorithmic performance, but also on workflow compatibility, clinician trust, reimbursement pathways, regulatory readiness, and evidence that these tools improve diagnostic confidence, efficiency, or patient outcomes.

#### Closing Perspective

Taken together, the future of ultrasound radiomics lies not simply in generating more features, but in building standardized, validated, and clinically actionable systems. Progress in standardization, external validation, workflow integration, AI-enabled automation, and regulatory readiness will determine whether ultrasound radiomics remains primarily a research tool or evolves into a practical component of precision imaging. The field is still in a developmental stage, but its trajectory suggests substantial potential for meaningful clinical impact.

## 7. Conclusions

Ultrasound radiomics is an emerging quantitative approach that may enhance pediatric ultrasound by providing objective biomarkers of tissue heterogeneity, disease classification, and longitudinal change beyond conventional visual assessment. Current evidence across brain, liver, renal, oncologic, lung, and musculoskeletal applications is encouraging, but remains limited by small retrospective single-center studies, heterogeneous acquisition protocols, variable segmentation methods, limited reproducibility testing, and insufficient external validation. Future progress will require standardized acquisition and analysis workflows, multicenter prospective validation, robust reproducibility assessment, and clinically interpretable tools that integrate smoothly into pediatric ultrasound practice. With these advances, ultrasound radiomics may evolve from an exploratory research method into a practical component of precision pediatric imaging.

## Data Availability

No new data were created or analyzed in this study. Data sharing is not applicable to this article.
